# Supplementary material for: Quantification of the Chemical Chaperone 4-Phenylbutyric Acid (4-PBA) in Cell Culture Media via LC-HRMS: Applications in Fields of Neurodegeneration and Cancer
Source: Pharmaceuticals (Basel). 2023 Feb 14;16(2):298. doi: 10.3390/ph16020298 (PMC9960049; doi:10.3390/ph16020298)
Supplement: Supplementary file 1 [file pharmaceuticals-16-00298-s001.zip › pharmaceuticals-2084776-supplementary.pdf]

Supplementary Material

Table S1. Instrumental LC-HRMS settings. Representative scheme of the LC-HRMS method used to quantify 4-PBA, branched in ESI and LC conditions. The mass recording settings of SIM (Selected Ion Monitoring) and PRM (Parallel Reaction Monitoring) include the following m/z ions, in which MS1 and MS2 stand for precursor and product ions, respectively.

**Tune data – ESI source**	**LC conditions**
• ESI- ionization<br>• Spray voltage: 2.80 kV<br>• Capillary temp.: 300 °C<br>• Sheath gas flow (N_2): 45.00 L/min<br>• Sweep gas flow (N_2): 0.00 L/min<br>• Aux. gas flow (N_2): 10.00 L/min<br>• Aux gas temp.: 300 °C<br>• Source Current: 1.80 µA	• Column: Synergi 4 µm Polar-RP 80 Å, LC 150 x 2 mm<br>• Column Temp.: 40 °C<br>• Mobile phases (UHPLC grade): A – H_2O 0.1% FA, B - MeOH 0.1% FA<br>• Flow rate: 0.300 mL/min<br>• Gradient elution: A-B (40:60, v/v) for 5.0 min, A-B (0:100, v/v) in 0.1 min, A-B (0:100, v/v) from 5.1 to 8.0 min, A-B (40:60, v/v) from 8.1 to 10.5 min<br>• Total run time: 10.5 min<br>• Inj. volume: 5 µL
• **Instrument**: Q-Exactive Plus UHMR Hybrid Quadrupole Orbitrap™ Mass Spectrometer equipped with a Vanquish™ Duo UHPLC system (Waltham, MA, USA)	

Analyte	MW [g/mol]	Ion mode	MS1 [m/z]	MS2 [m/z]	Collision Energy [eV]
4-PBA	164.20108	ESI-	163.07645	91.05528	65
NPBA	209.19864	ESI-	208.06153	136.04053	65
TG	650.75360	ESI-	649.32295	87.04515	65

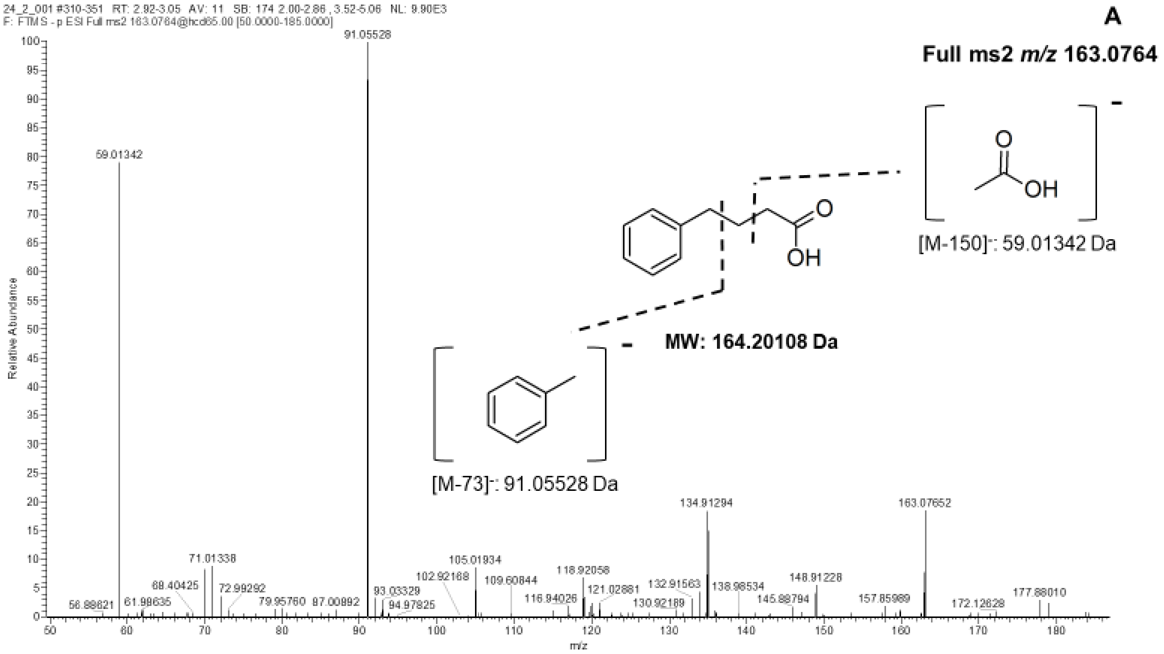

A
Full ms2 m/z 163.0764
[M-150]⁻: 59.01342 Da
MW: 164.20108 Da
[M-73]⁻: 91.05528 Da
Relative Abundance
m/z

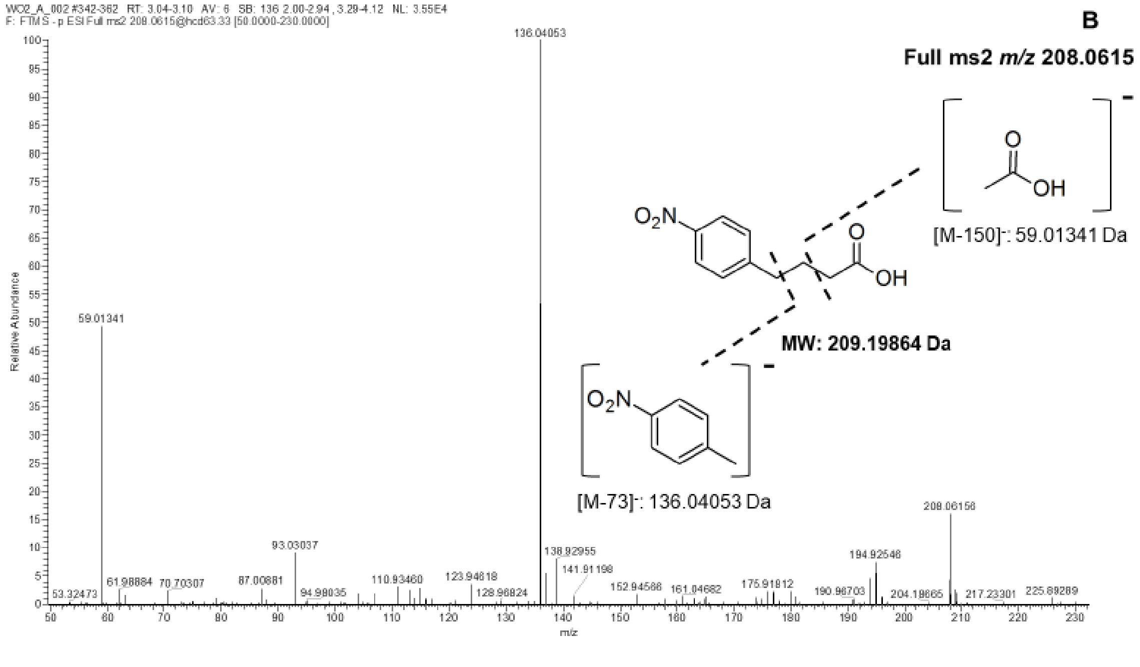

B
Full ms2 m/z 208.0615
[M-150]⁻: 59.01341 Da
MW: 209.19864 Da
[M-73]⁻: 136.04053 Da
Relative Abundance
m/z

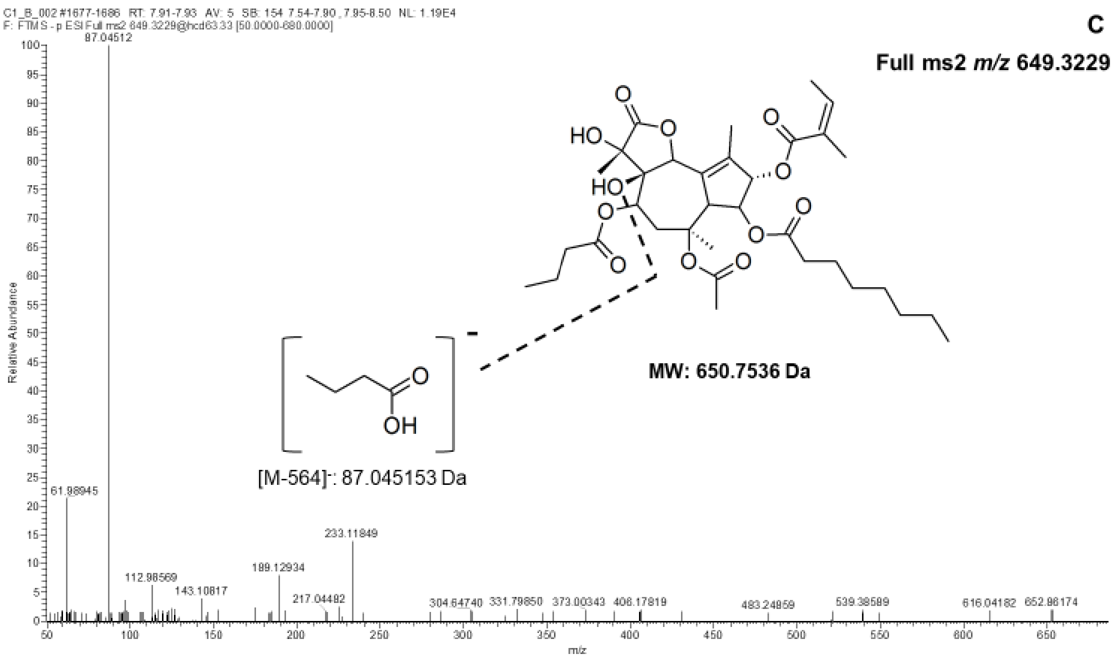

Figure S1. Analytes and IS mass spectra.

(A). MS/MS spectra of 4-PBA precursor ion. (A) Precursor ion fragmentation of 4-PBA showed product ion peaks with masses of [M-73]- and [M-150]-. (B). MS/MS spectra of NPBA precursor ion. (B) Interestingly, precursor ion of NPBA displayed a fragmentation related to 4-PBA, since there are still product ion peaks with masses of [M-73]- and [M-150]-. (C). MS/MS spectra of TG precursor ion. (C) Precursor ion fragmentation of thapsigargin (TG) figured an intense product ion peak with a mass of [M-564]-.

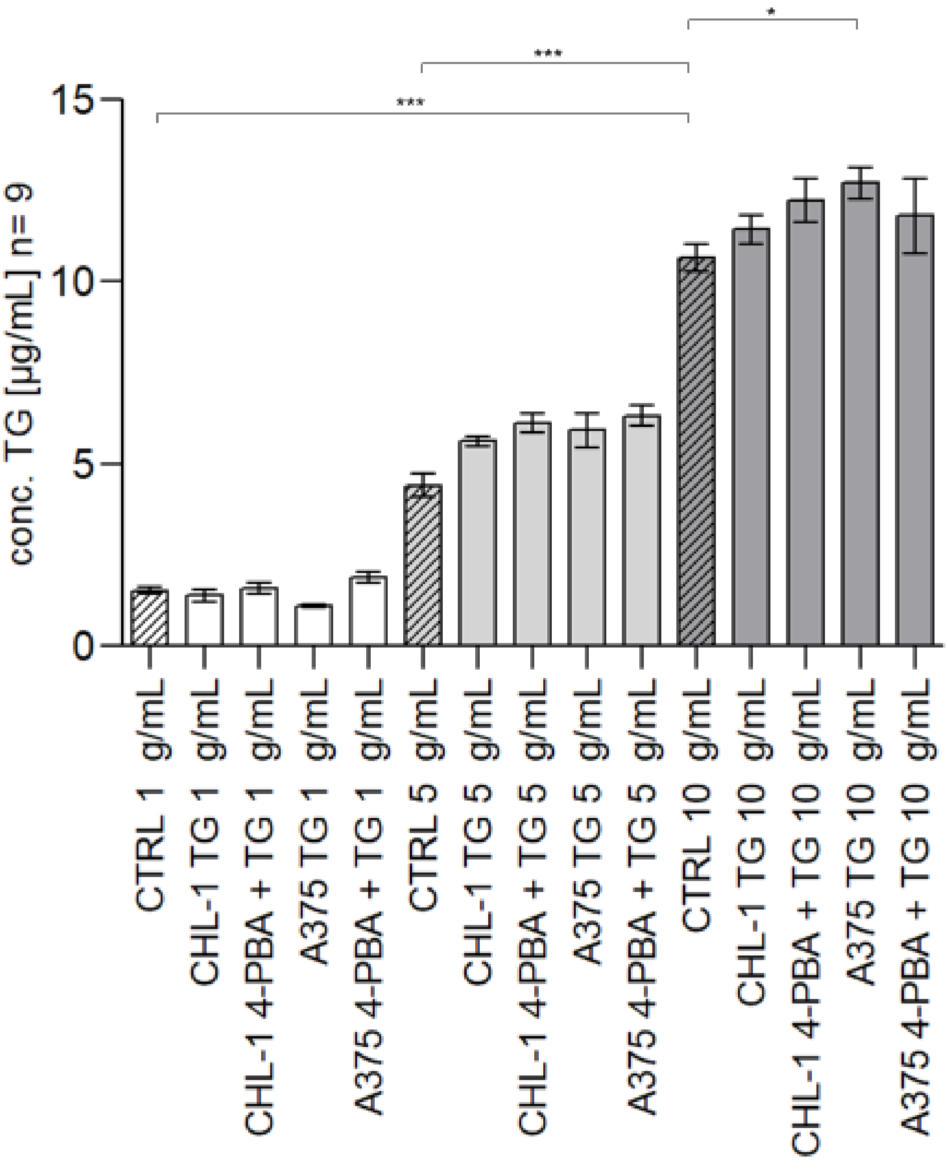

Figure S2. Monitoring of operative TG concentration by semi-quantitative LC-HRMS. Concentrations of TG were evaluated in CHL-1 and A375 conditioned cell culture media at 24h, by LC-HRMS analysis. TG initial treatment concentrations at initial time (t0) were used as an internal control (CTRL). TG concentrations were expressed as µM ± SE. Experiments were performed in triplicate and repeated three times (* p< 0.05; ** p<0.01; *** p<0.001; by one- way Anova, Sidak's multiple comparison).

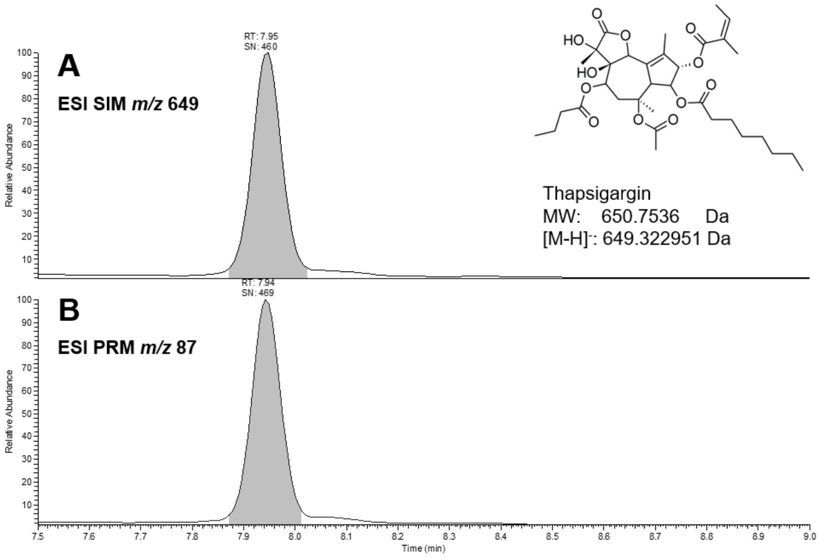

Figure S3. Chemical structure and chromatograms of thapsigargin (TG). (A) and (B) show the chromatograms recorded in SIM and PRM acquisition modes, respectively.

Supplemental S1. Details for a general bioanalytical validation for ICH, FDA and EMA guidelines.

It is possible to find all the recommendations for the bioanalytical validation on these links.

a. https://www.ema.europa.eu/en/documents/scientific-guideline/guideline-bioanalytical-method-validation_en.pdf

b. https://www.fda.gov/files/drugs/published/Bioanalytical-Method-Validation-Guidance-for-Industry.pdf

c. https://www.ema.europa.eu/en/documents/scientific-guideline/ich-guideline-m10-bioanalytical-method-validation-step-5_en.pdf
